# Effects of Soil Compaction Stress Combined with Drought on Soil Pore Structure, Root System Development, and Maize Growth in Early Stage

**DOI:** 10.3390/plants13223185

**Published:** 2024-11-13

**Authors:** Xiangming Zhu, Wei Peng, Qingyang Xie, Enhua Ran

**Affiliations:** State Key Laboratory of Black Soils Conservation and Utilization, Northeast Institute of Geography and Agroecology, Chinese Academy of Sciences, 138 Haping Road, Harbin 150081, China; apengwei0522@163.com (W.P.); xieqingyang24@mails.ucas.ac.cn (Q.X.); ranenhua22@mails.ucas.ac.cn (E.R.)

**Keywords:** maize, soil compaction, drought, soil pore characteristics

## Abstract

Soil compaction is a major environmental stress to root development and plant growth. Meanwhile, drought always results in increasing soil mechanical impedance, which in turn aggravates soil compaction stress. In this study, a column experiment with three levels of compaction stress (low, moderate, and severe) and two levels of soil water content (well-watered and drought,) was established to investigate the effects of soil compaction combined with drought on soil pore structure, root development, and maize growth properties. The results showed that soil compaction combined with soil water stress significantly affected the characteristics of soil pore structure. With the increase in soil compaction, the porosity, larger pores (>500 μm), and maximum pore diameter significantly decreased (*p* < 0.05) regardless of soil water status. Additionally, both pore morphology and network parameters also deteriorated under soil compaction with drought conditions. Soil compaction substantially affected the root length, root volume, root surface area, and root average diameter in the whole profile (*p* < 0.05). Compared to well-watered conditions, the effects of soil compaction on root characteristics under drought conditions were more obvious, which indicated that appropriate soil water content could alleviate compaction stress. The aboveground biomass and plant height showed a consistent trend with root traits under soil compaction stress regardless of water status. A Pearson’s correlation analysis showed that there were significant correlations between most soil pore parameters and maize growth traits. In addition, soil compaction showed a significant effect on both stomatal conductance and transpiration rate while soil water showed a significant effect on SPAD (Soil Plant Analysis Development).

## 1. Introduction

Soil compaction is one of the physical forms of soil degradation, which plays a critical role in many soil-related problems such as soil erosion, nutrient depletion, pollution, and greenhouse gas emissions [1,2]. Compaction in agricultural systems is often caused by heavy machinery use, livestock or human trampling, and ineffective irrigation or tillage [3]. Soil compacting in global farmlands has become a major problem because of its detrimental effects on the environment and agricultural sustainability [4]. It is estimated that soil compaction has affected about 68 million ha of the world’s farmlands [5], and among them more than 32% of European subsoils are reported to be compacted [6]. Given the demand for food escalating globally, it is critical to comprehend how soil compaction affects plants.

The most obvious effect of soil compaction on soil physical properties is a reduction in porosity and an increase in soil bulk density and mechanical impedance, which ultimately leads to deterioration of the soil structure [7]. Soil pores are the main channel of water movement and root elongation. Therefore, an analysis of the pore structure characteristics of plants is necessary to comprehend the impacts of soil compaction on plant growth [8].

In agricultural systems, increased mechanical impedance profoundly affects root growth and root structure. Root structure determines soil exploration and exploitation within the soil profile, and has a major impact on soil water and nutrient uptake, stress tolerance, and crop productivity [7,9]. Many previous studies have shown that high soil compaction stress leads to root morphological modification, such as a decrease in the size of the root system (including changes in root number and length) and a lower root elongation rate [10], in addition to swollen, circular, or flattened root tips [11], smaller angular spread [12], and altered branching patterns depending on the plant species [13]. Meanwhile, the effect of soil compaction on root growth depends on the degree of compactness and soil water status. Under moderate compaction conditions, the effect of soil compaction may be positive. For example, Bouwman [14]. observed that soil compaction increased crop yield. Such discrepancies reflect that the relationship between soil compaction and root growth remains unclear.

Soil compaction not only affects root growth, but also restricts shoot performance. It was reported that the aboveground dry weights of *Scutellaria baicalensis* decreased by 22.35% under soil compaction conditions with 1.5 g cm^−3^ bulk density [15]. The root to shoot ratio, as well as the tiller numbers of some cereal crops such as rice can decrease under compaction conditions [9,16]. Aboveground plant growth is impacted as leaf elongation rate can be reduced [17]. and the rate of leaf appearance decreases [12,18]. when roots experience soil compaction stress. Furthermore, there is significant variation in how different species and cultivars react to compacted soil. Research has indicated that the emergence of soybean, barley, and maize seedlings is more sensitive to soil compaction [19]. In the field, soil strength increases rapidly as soil dries [16]. The impact of drought on plant growth has been extensively studied; however, the coupling impact of soil strength and drought remains unclear. Therefore, revealing the effects of soil compaction combined with drought on plant growth will provide guidance for improved technologies and methods to alleviate soil compaction-induced plant damage.

Soil compaction effects extend beyond root and shoot morphology, affecting general plant physiology. Several authors have reported a decrease in photosynthetic activity due to a drop in stomatal conductance in plants grown on compacted soils [20,21]. Other responses of plants to those stresses are changes in tissues’ water content, membrane permeability, and chlorophyll content [22]. Plants adapt to hypoxia by metabolic processes such as maintaining carbohydrate content, avoiding acidification of the cytoplasm, and launching a defense antioxidant system.

Hence, the aims of this study were as follows: (1) to quantitatively assess the effects of soil compaction combined with drought on soil pore structure parameters; (2) to clarify the effects of soil compaction combined with drought on root system development and maize growth; and (3) to explore the potential relationship between soil pore structure and maize growth under soil compaction and soil water stress. This study will provide valuable insights into the combined effects of soil structure and maize growth in response to both soil compaction and drought stresses and contribute to optimizing soil tillage and water management in the field.

## 2. Results

### 2.1. Soil Pore Characteristics

**Figure 1:**
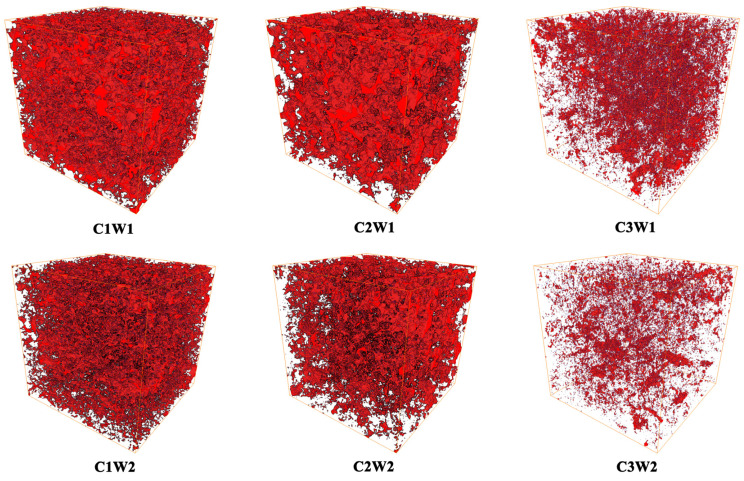
Representative 3D images of the soil cores under different treatments. Note: in the 3D pore structure images, red represents soil pores.

Figure 1 illustrates representative samples of the 3D pore structure of different treatments. As shown in Figure 1, the number of soil pores decreased significantly with increasing soil bulk density under the same soil water status, and increased significantly with increasing soil water content under the same bulk density. All the soil pores were irregular and had a lack of connectivity due to all the columns packed with the sieved soils. The specific differences in pore characteristics among the treatments are detailed in Table 1. Under well-watered conditions (W1), the C1W1 treatment exhibited significantly higher *ϕ*, CLP, MAPD, and *Γ* values, while exhibiting a lower CP value than the C3W1 treatment (*p* < 0.05). However, there was no significant difference in HR, DA, and SSA among different soil compaction stress treatments. A similar trend was found under drought conditions (W2). Under the same compaction stress, the means of porosity, CLP, and MAPD were higher under well-watered conditions than under drought conditions. There was no significant interaction between C and W in terms of all soil pore characteristic parameters (*p* > 0.05) (Table 1).

**Figure 2:**
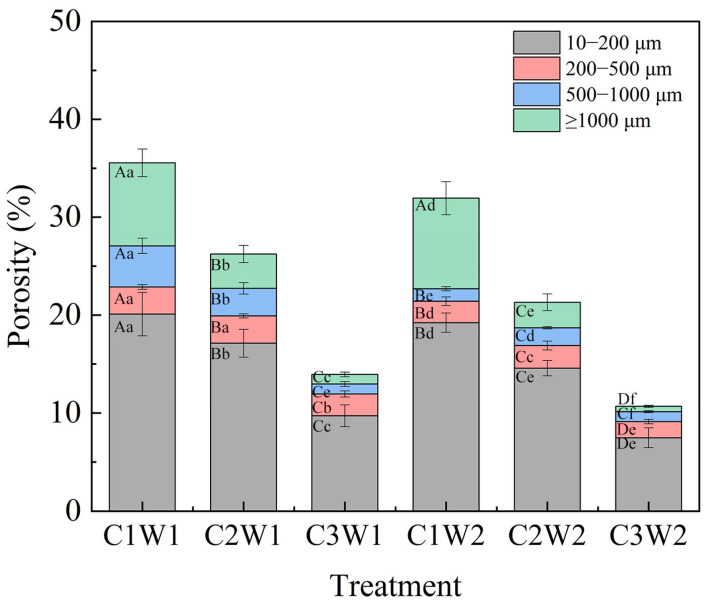
Pore size distribution of soil pores under different treatments. Note: Different letters indicate significant differences among treatments (*p* < 0.05). The capital letters in the same column represent significant differences (*p* < 0.05) at different compaction levels under the same water condition. The small letters in the same column represent significant differences (*p* < 0.05) at different soil water status under the same compaction level.

Soil pore size distributions under different treatments are shown in Figure 2. In terms of compaction stress, for 10–200 μm, 500–1000 μm, and > 1000 μm pore classes, the porosity of all treatments decreased with increasing soil bulk density regardless of soil water status. Regarding the 200–500 μm pore class, there were no significant differences between C1W1 and C2W1. In terms of soil water conditions, the porosity of 500–1000 μm and > 1000 μm under well-watered conditions was significantly higher than those under drought conditions (*p* < 0.05).

### 2.2. Biomass Accumulation and Plant Height

**Figure 3:**
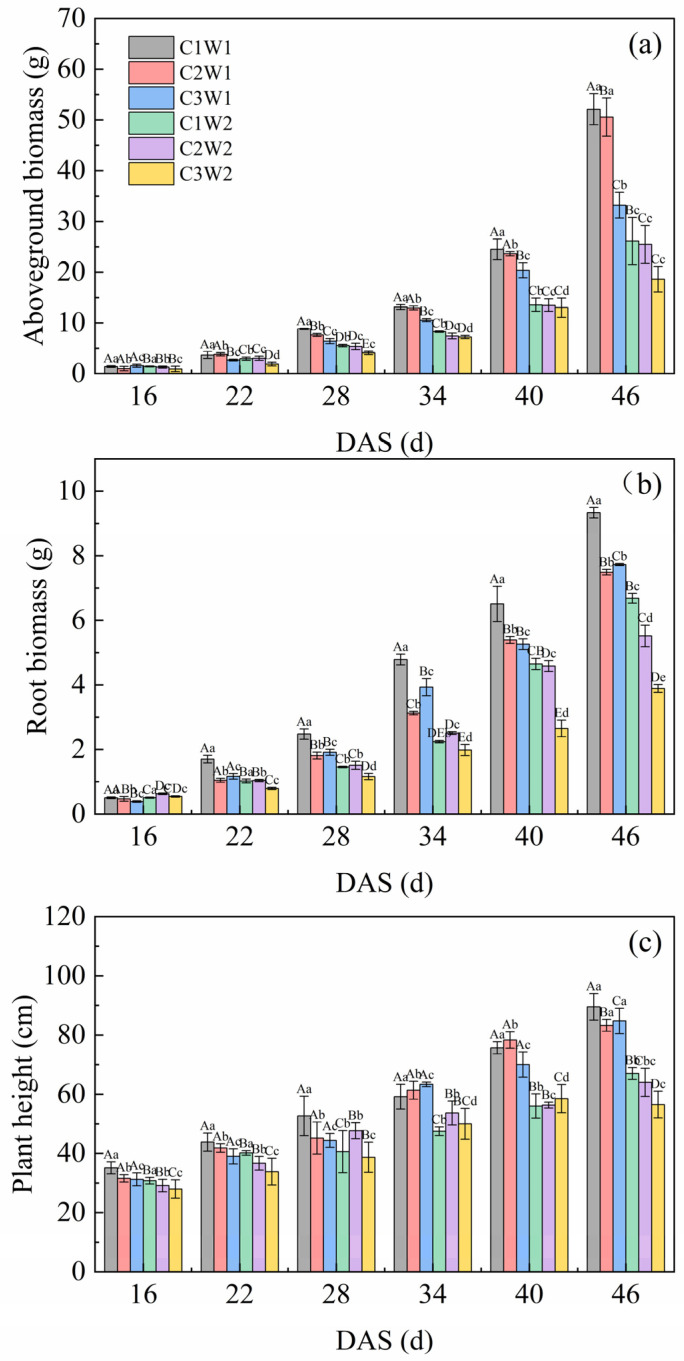
Dynamic changes in the (**a**) aboveground biomass, (**b**) root biomass, and (**c**) plant height of maize during the entire growth period. Note: Different letters indicate significant differences among the treatments (*p* < 0.05). The capital letters in the same column represent significant differences (*p* < 0.05) at different compaction levels under the same water condition. The small letters in the same column represent significant differences (*p* < 0.05) at different soil water status under the same compaction level.

As presented in Figure 3, compaction stress combined with soil water stress generally had a significant impact on aboveground biomass, root biomass and plant height throughout the growth period. For aboveground biomass, the C1W1 and C2W1 treatments had significantly higher values than that of the other treatments from 22 DAS to 46 DAS (*p* < 0.05). The order of the aboveground biomass values under all treatments was C1W1 > C2W1 > C3W1 > C1W2 > C2W2 > C3W2 from 28 DAS to 46 DAS (Figure 3a). Similar to the aboveground biomass, the root biomass of C1W1 was also highest among all treatments (Figure 3b). The root biomass of the treatments under well-watered conditions was significantly higher than those under drought conditions (*p* < 0.05). For plant height, there were generally no significant differences among the different compaction stress treatments under the same soil water status throughout the growth period (Figure 3c).

### 2.3. Leaf Photosynthetic Characteristics

Under well-watered conditions, compaction stress gradually decreased both stomatal conductance (Gs) and transpiration rate (Tr), which were 59.26% and 60.00% lower under the C3W1 treatment than under the C1W1 treatment (Table 2). In terms of soil water stress, the SPAD values of the treatments under well-watered conditions were generally higher than those under drought conditions. Soil water stress did not show significant effects on either Gs or Tr, and there were no significant interactions between soil compaction and soil water content.

### 2.4. Root Structure Characteristics

Overall, the ANOVA results (Table 3) showed that both soil compaction and soil water content had significant differences in root length, root surface area, root volume, and root average diameter. Specifically, the means of the root surface area and root length in the C1W1 treatment decreased by 9.87% and 15.50% in comparison to those in the C2W1 treatment, and significantly decreased by 16.50% and 26.94% in comparison to those in the C3W1 treatment (*p* < 0.05). Additionally, root average diameter in the C1W1 treatment was significantly lower than that in the C3W1 treatment (*p* < 0.05). There was a significant interaction between C and W in terms of root average diameter. A similar trend was also found under drought conditions (W2). With the increase in compaction stress, root surface area, root volume, and root length significantly decreased, and root average diameter significantly increased (*p* < 0.05).

**Figure 4:**
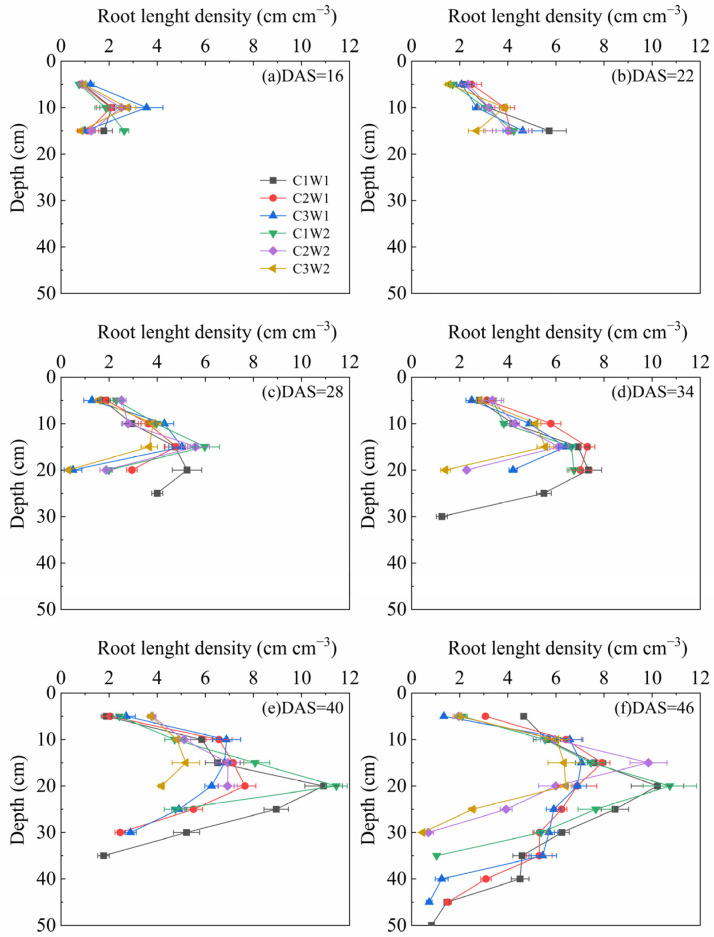
Dynamic changes in the root length density of maize during the entire growth period.

Root length density is a crucial metric in water and nutrient uptake. The dynamic change in root length density under different compaction and soil water content stress from 16 DAS to 46 DAS is shown in Figure 4. The distribution of root length density among treatments was similar and the rooting depth of treatments under well-watered conditions was basically greater than those under drought conditions. Furthermore, the root length density of the C1W1 and C3W2 treatments were the highest and the lowest among all the treatments during most growth stages in the soil profile, which was consistent with both root biomass and root length.

### 2.5. Relationship Between Soil Pore Characteristics and Maize Growth Characteristics

**Figure 5:**
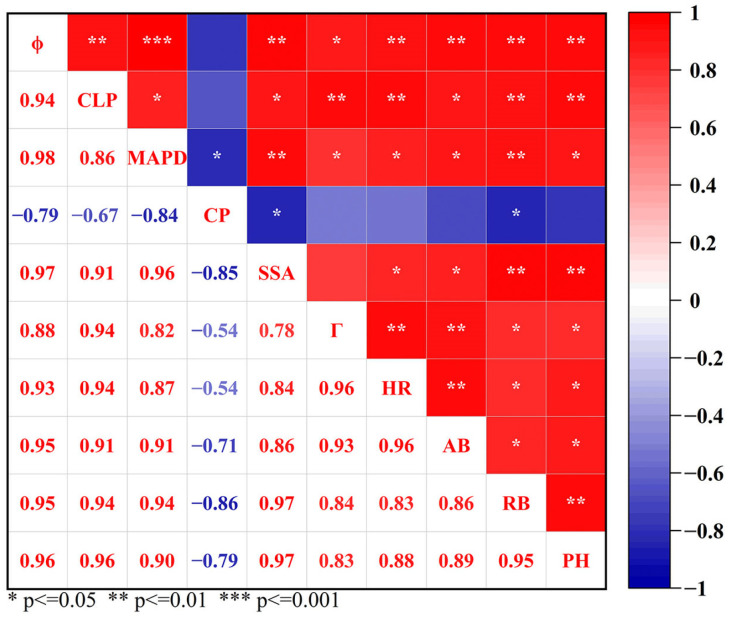
Correlation analysis among the parameters of soil pore, maize growth, and soil water storage. *ϕ*: porosity; HR: hydraulic radius; DA: degree of anisotropy; CLP: connected largest porosity; MAPD: maximum pore diameter; CP: compactness; *Γ*: global connectivity; SSA: specific surface area.

The Pearson’s correlation analysis showed that there were significant correlations between most soil pore parameters and maize growth traits (Figure 5). Among them, *ϕ*, CLP, MAPD, SSA, *Γ*, and HR were all significantly positively correlated with root biomass (RB), but CP was negatively correlated with RB (*p* < 0.05). In addition, CP was not significantly correlated with aboveground biomass (AB) and plant height (PH) (*p* > 0.05). In addition, there was an obvious correlation between the soil growth traits.

## 3. Discussion

### 3.1. Effects of Soil Compaction Stress Combined with Drought on Soil Pore Structure

Soil pores play a critical role in many soil processes and functions, such as water permeability, gas diffusivity, and microbial activity [23,24]. Soil compression is usually accompanied with a decrease in porosity and changes of pore characteristics [23]. Our study confirmed this point, especially under drought conditions. In this study, with an increase in soil bulk density, the porosity significantly decreased from 31.97% to 10.66% under drought conditions (Table 1), where 10% was generally assumed to be the critical value for root elongation and crop growth [10,25]. From the 3D visualization of the XCT-detected pore systems, we also could clearly find that the soil pores decreased with the increase in bulk density (Figure 1). These observation aligned with many previous studies that have reported the impact of compaction on soil pore characteristics [26,27]. In addition, the porosity of the W1 treatment was a bit higher than that of the W2 treatments under the same compaction stress conditions (Table 1). This might be due to the fact that the W1 treatments had more root elongation than the W2 treatments (Table 3, Figure 4).

We observed that the decrease in porosity with compaction stress was mainly in larger pores (>500 μm) (Figure 2). For example, the ratio of larger pores (>500 μm) to total pores decreased from 36.24% under the C1W1 treatment to 14.28% under the C3W1 treatment. This result was consistent with a soil column experiment by Colombi et al. [28], who also found that larger pores were susceptible to soil compaction. Besides porosity and pore size distribution, the effects of soil compaction on pore morphology and network properties were also significant. The values of DA in the soil columns were around 0.25 (Table 1), indicating that the pores were anisotropic in all directions. The hydraulic radius, compactness, and fractal dimension showed a significant increase with the increase in compaction stress. These results indicated that bulk density played a vital role in modifying soil macropore morphology. Soil pores are a complex network of interconnected, rather than isolated, voids, with different shapes and sizes [29]. Our findings showed that the global connectivity, connected largest porosity, and specific surface area were all the largest in the C1W1 treatment (Table 1). This result implied that proper compaction and sufficient soil water content enhanced the connectivity of pores.

### 3.2. Effects of Soil Compaction Stress Combined with Drought on Root System Development

Roots are critical for plants to acquire water and nutrients from soil. Soil compaction and water content are two important factors for root system development. It is widely reported that rooting depth and root length are generally limited by a combination of mechanical impedance and water stress [13,25]. In our study, the rooting depth was greatly reduced under severe compaction and drought stress (Figure 4), which was in agreement with many other observations [30]. Compaction altered root length distribution, generally shifting root length distribution to shallower layers (Figure 4). Multiple studies have described similar redistributions of roots under impeded field conditions for various crops including maize [30,31]. Furthermore, root biomass showed an overall decreasing trend with the increase in soil bulk density whether under well-watered conditions or under drought conditions (Figure 3b). One possible reason is that soil compaction modified the pore size and porosity and decreased the unsaturated hydraulic conductivity substantially, which ultimately confined root growth [32]. The significant correlations between the soil pore characteristic parameters and root biomass further confirmed this inference (Figure 5).

Besides root length density and rooting depth, the effect of soil compaction stress combined with drought on root morphological traits was also significant (Table 3). Our results show that the root surface area and root volume decreased gradually with the increase in soil bulk density, especially under drought conditions, which was in compliance with the findings of Lipiec et al. [11]. On the contrary, the root average diameter increased with increasing compaction stress, especially under drought conditions. Root cell deformation and blurring of cell boundaries were seen when soil bulk density increased [25]. The compaction of the soil caused these alterations in the root morphology, which restricted the plant’s ability to absorb nutrients and water from the soil [33].

### 3.3. Effects of Soil Compaction Stress Combined with Drought on Maize Growth

In this investigation, we discovered that soil compaction had a discernible detrimental impact on aboveground biomass (Figure 3), particularly following 28 DAS. Similarly, Tubeileh et al. [32] also found that the shoot biomass and shoot to root ratio were lower in the case of soil compaction stress. However, in our study, plant height was mainly affected by soil water status. This might be attributed to the large differences of water supply between W1 and W2. Although plant species can vary in their sensitivity to drought, one of the main plant responses to it is a substantial reduction in shoot biomass but a moderate reduction in root biomass [34]. Additionally, the response of cereal species to the combined effect of different soil compaction conditions with drought depends on the level of soil compaction and soil water content [26].

Photosynthesis is the most basic life activity of plants, and it is one of the physiological processes most sensitive to abiotic stress [35]. Ripley et al. [22] found that the first response of plants to soil compaction and drought are changes in the tissues’ water content, chlorophyll content, and gas exchange parameters. Our results also showed that the stomatal conductance (Gs) and transpiration rate (Tr) decreased significantly with the increase in compaction regardless of soil water status (Table 2). Soil water content had a significant effect on SPAD values. These results are similar to those of Tubeileh et al. [32].

## 4. Materials and Methods

### 4.1. Soil Column Experiment

The soil was sampled from 0 to 20 cm soil layer under a continuous maize cropping system at Hailun (47°26′ N, 126°47′ E), Heilongjiang Province in Northeast China. The soil is typical black soil (Haplic phaeozems in the World Reference Base for Soil Resources classification), composed of 67.62 g kg^−1^ organic matter, 1.83 g kg^−1^ total nitrogen, 0.74 g kg^−1^ total phosphorus, and 17.29 g kg^−1^ total potassium, with pH 6.79. The particle-size distribution of the soil included 14.2% of sand, 59.4% of silt, and 26.4% of clay. The soil was air-dried and sieved to <2 mm for the column experiment. In all, 120 PVC columns measuring 53 cm in height and 20 cm in diameter were employed in the experiment. PVC back coverings were used to seal the bottom of each column. In this study, we considered two factors: compaction stress and soil water content. Considering the Proctor reference bulk density of the sampled soils was about 1.61 g cm^−3^ and the local bulk density after harvest was about 1.24 g cm^−3^, three levels of bulk density (i.e., 1.1, 1.3, and 1.5 g cm^−3^) were chosen to represent low, moderate and severe compaction (hereafter referred to as C1, C2, and C3, respectively). Soil columns were packed with air-dried soil at every 5-cm layer according to the calculated weight, and after compacting each layer, the soil surface was scraped to guarantee homogeneous packing.

**Figure 6:**
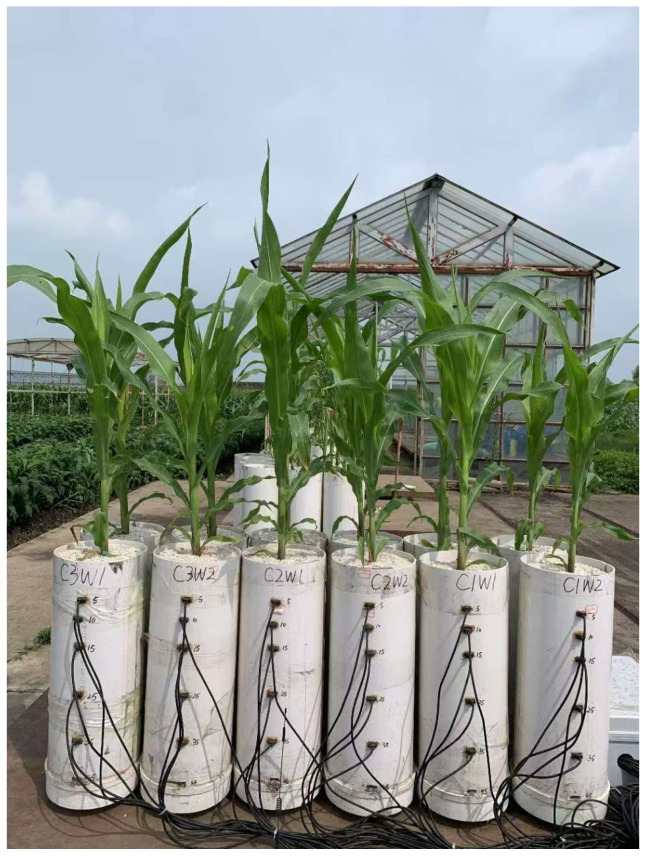
Experimental devices and soil water content monitoring.

Maize seeds (*Demeiya3*) were germinated on wet filter paper at 30 °C for 48 h and then planted 3 mm below the soil surface in the columns with a seed density of one plant per column. The experiment lasted for 46 d (from 1 June 2022 to 16 July 2022) from sowing to jointing stages of maize. At the soil surface of each column, 3 cm of fine quartz sand was filled to reduce soil surface evaporation on 10 June 2022 (10 days after sowing, 10 DAS). Until 16 June 2022 (16 DAS), all the seedlings in the columns were irrigated sufficiently. Two soil water content treatments (referred to as W1 and W2, representing well-watered and drought conditions), were set on 16 DAS with maize irrigated every 6 days using different amount of water. The W1 treatments were maintained with an average soil water content in the root zone of no less than 80% of field water capacity by weighting. The irrigation volume for the W2 treatments was half that of the W1 treatments. Together with the compaction treatments, this study involved 6 treatments: C1W1, C2W1, C3W1, C1W2, C2W2, and C3W2. One column for each treatment was selected to install EC-5 sensors (Meter Group Inc., Pullman, WA, USA) at a depth of 5, 10, 15, 25, and 35 cm from the soil surface to monitor volumetric soil water content of the columns (Figure 6). All the columns were placed under natural conditions with a rain shelter and arranged in a completely random manner.

From 16 DAS to 46 DAS, sampling work was done every 6 days and a total of 6 times. At each sampling time, three columns were selected randomly to cut down the shoots of maize and opened to sample roots. The shoots collected from each seedling were measured for plant height and then dried for 48 h to a constant weight at 70 °C and weighed. The opened soil columns were cut into 5-cm soil layers from the soil surface to rooting depth. The soil of each layer was put into a mesh with grids of 0.05 cm in diameter, the soil was carefully washed away, and the roots in each soil layer were picked out. The collected roots were then scanned with a scanner (Snapscan 1236, AGFA, Dusseldorf, Germany), and analyzed with the WinRHIZO Pro software package (Version 2009b; Regent Instruments Inc., Quebec, QC, Canada) for root morphological traits. The dry weight of roots was also measured in the same way as shoots. In addition, three plants of each treatment were selected to measure leaf photosynthesis indexes and chlorophyll content index (SPAD) on 46 DAS. According to Ren et al. [36], the photosynthetic indexes including stomatal conductance (Gs) and transpiration rate (Tr) were measured at 9:00~11:00 a.m. on a sunny day by Li-600 portable photosynthesis system (LI-COR, Lincoln, NE, USA). During the measurement, three uniform plants for each treatment were selected and the third and fourth leaves from the top were used for measurement. To determine the leaf SPAD value, the SPAD values of three fully unfolded leaves from the top were recorded using a SPAD-502 chlorophyll meter (Minolta Camera Co., Osaka, Japan) [37].

To investigate the soil pore structure of each treatment, the intact soil cores were collected from the upper layer using PVC cylinders (6 cm in height, 5 cm in inner diameter) on 46 DAS. After carefully pressing the PVC cylinders into the ground, the surrounding material was gradually removed. After that, the retrieved soil cores were wrapped in plastic films right away to stop any water loss and carefully moved to the laboratory without causing any disturbance. Prior to measurements, the soil cores were kept at 4 °C in a refrigerator. Eighteen intact soil cores were obtained by triplicate replication of each treatment. 

### 4.2. XCT Imaging and Data Processing

The soil cores were scanned using industrial X-ray computed tomography (GE, Sensing and Inspection Technologies, GmbH, Wunstorf, Germany) at an energy level of 80 kV and a current is 100 μA. The digital image processing and quantification of soil pore characteristics were done using ImageJ software (Vs. 1.49 s, National Institutes of Health, http://imagej.nih.gov/ij/) [23]. To remove the PVC wall effect, a region of interest (ROI) of 30 mm in diameter and 30 mm in height was carefully selected from the central part. After reducing image noise with a median filter (2 × 2 × 2 pixels), image segmentation was performed using auto locally adaptive segmentation method to identify soil pore and matrix. Subsequently, image analysis was conducted using the processed binary images. The 3D pore structure images were visualized using the ImageJ Bone-J plugin [38].

The porosity (*ϕ*) was defined as the percentage of the CT-derived pore (>10 μm) volume to the total volume of the ROI [39]. In this study, the hydraulic radius (HR), compactness (CP), degree of anisotropy (DA), connected largest porosity (CLP), global connectivity (*Γ*), specific surface area (SSA), and maximum pore diameter (MAPD) of soil pores were employed to represent pore characteristics. HR was computed as the ratio of the volume and surface area of the macropores in the soil. The larger the hydraulic radius, the greater the capacity of water and air conduction [39]. CP was a pore shape factor, whose value increased as the pore deviated more from a sphere [40]. The porosity roundness was represented by DA, and the closer DA was to zero, the closer the porosity was to the circle. Pore morphology was also more regular [41].

The volume of pores was calculated using the “particle analyzer” by the Bone-J plugin. CLP was the volume of the largest interconnected macropore network in the soil as a percentage of the ROI volume [41]. Before connectivity analysis, the binary image should be applied “purify” in the Bone-J plugin to obtain the largest interconnected macropores network [42,43]. It was measured using the “particle analyzer” plugin in ImageJ. SSA was the ratio of pore surface area to total volume of the ROI. When the SSA of pores was larger, the connectivity of pores would also increase, which was conducive to hydraulic conduction [39]. MAPD was obtained by using “Thickness” function in Bone-J plugin. In this study, four pore size classes (10–200 µm, 200–500 µm, 500–1000 µm, and >1000 µm) were chosen based on the variation trends of pore size distributions among the treatments [44].

### 4.3. Statistical Analysis

The statistical analysis was performed using IBM SPSS Statistical 26.0 (IBM, New York, NY, USA). The effects of compaction stress combined with drought on soil pore structure, root system development, and maize growth were analyzed by two-way ANOVA. The least significant difference (LSD) for statistical significance of *p* < 0.05 was calculated by comparing the means. To determine exactly which variants had statistically significant differences, Duncan’s test was used. Pearson’s correlation analysis was employed to evaluate the relationship between soil pore characteristics and maize growth parameters with 95% confidence (α = 0.05).

## 5. Conclusions

Our study confirmed that soil compaction combined with soil water stress significantly affected the characteristics of soil pore structure, root system development, and maize growth under our soil column experiment. At a given soil water status (drought or well-watered), soil bulk density significantly affected the porosity, larger pores (>500 μm), and maximum pore diameter (*p* < 0.05). In addition, pore morphology and network parameters (including HR, CP, DA, *Γ*, CLP, etc.) also deteriorated significantly under soil compaction conditions, especially combined with drought. Soil compaction combined with drought consistently resulted in decreased root length, root surface area, root volume, and root surface area, as well as shoot biomass and root biomass. However, no significant interaction was found in most root structure traits between soil compaction and water content, with the exception of root average diameter. Furthermore, soil compaction showed a significant effect on both stomatal conductance and transpiration rate while soil water showed a significant effect on SPAD. The significant correlations between most soil pore parameters and maize growth traits suggested that proper bulk density and soil water status was critical to plant growth. These results will provide a better understanding of the effects of soil compaction on soil pore structure and plant growth. Future experimental field work is essential to determine whether similar results will be achieved by soil compaction combined with drought.

## Figures and Tables

**Table 1:** Characteristics of total pores derived from XCT images.

Treatments	*ϕ* (%)	HR (mm)	DA	CLP (%)	MAPD (mm)	CP	*Γ*	SSA (m^−1^)
C1W1	35.25 ± 0.42 Aa	0.015 ± 0.004 Aa	0.22 ± 0.03 Aa	28.69 ± 0.32 Aa	0.39 ± 0.03 Aa	9.43 ± 0.43 Ba	0.04 ± 0.008 Aa	787.49 ± 7.03 Aa
C2W1	26.23 ± 0.32 Bb	0.014 ± 0.004 Ab	0.23 ± 0.02 Ab	19.67 ± 0.22 Bb	0.33 ± 0.03 ABb	10.27 ± 0.43 ABb	0.02 ± 0.005 Bb	781.96 ± 7.28 Ab
C3W1	13.93 ± 0.19 Cc	0.013 ± 0.003 Ac	0.25 ± 0.03 Ac	9.84 ± 0.13 Cc	0.26 ± 0.02 Bc	14.40 ± 0.65 Ac	0.01 ± 0.002 BCc	753.59 ± 7.88 Ac
C1W2	31.97 ± 0.35 Db	0.012 ± 0.003 Ba	0.23 ± 0.02 Ba	19.67 ± 0.23 Db	0.38 ± 0.03 Ca	8.56 ± 0.48 Da	0.008 ± 0.001 Db	781.63 ± 7.08 Ba
C2W2	21.31 ± 0.27 Dc	0.012 ± 0.002 Bb	0.23 ± 0.02 Bb	10.66 ± 0.14 Ec	0.33 ± 0.03 CDb	11.63 ± 0.53 Db	0.005 ± 0.001 Dc	768.36 ± 7.26 Bb
C3W2	10.66 ± 0.14 Ec	0.012 ± 0.003 Bc	0.24 ± 0.03 Bc	7.38 ± 0.09 Ec	0.23 ± 0.02 Dc	30.75 ± 0.13 Cd	0.006 ± 0.001 Dc	745.01 ± 7.53 Bc
C	*	NS	NS	*	*	*	*	NS
W	*	NS	NS	NS	NS	NS	NS	NS
C*W	NS	NS	NS	NS	NS	NS	NS	NS

Note: *ϕ*: porosity; HR: hydraulic radius; DA: degree of anisotropy; CLP: connected largest porosity; MAPD: maximum pore diameter; CP: compactness; *Γ*: global connectivity; SSA: specific surface area. The capital letters in the same column represent significant differences (*p* < 0.05) at different compaction levels under the same water condition. The small letters in the same column represent significant differences (*p* < 0.05) at different soil water status under the same compaction level. * indicates significant differences at *p* < 0.05. NS, not significant at *p* < 0.05.

**Table 2:** Stomatal conductance (Gs), transpiration rate (Tr), and chlorophyll content index (SPAD) under different treatments on 46 DAS.

Treatments	SPAD	G_S_ (mol m^−2^s^−1^)	Tr (mmol m^−2^s^−1^)
C1W1	37.9 ± 1.90 Aa	0.54 ± 0.07 Aa	0.05 ± 0.005 Aa
C2W1	35.8 ± 2.83 Ab	0.51 ± 0.11 ABb	0.04 ± 0.01 ABb
C3W1	39.7 ± 1.78 Ac	0.22 ± 0.06 Bc	0.02 ± 0.005 Bc
C1W2	33.5 ± 1.99 Ba	0.46 ± 0.29 Ca	0.04 ± 0.02 Ca
C2W2	35.2 ± 1.15 Bb	0.41 ± 0.11 Cb	0.03 ± 0.01 Cb
C3W2	35.3 ± 3.95 Bc	0.30 ± 0.19 Cc	0.03 ± 0.01 Cc
C	NS	*	*
W	*	NS	NS
C*W	NS	NS	NS

Note: The capital letters in the same column represent significant differences (*p* < 0.05) at different compaction levels under the same water condition. The small letters in the same column represent significant differences (*p* < 0.05) at different soil water status under the same compaction level. * indicates significant differences at *p* < 0.05. NS, not significant at *p* < 0.05.

**Table 3:** Root structure characteristics under different treatments on 46 DAS.

Treatments	Root Surface Area (cm^2^)	Root Volume (cm^3^)	Root Length (cm)	Root Average Diameter (mm)
C1W1	8974.3 ± 863.0 Aa	94.5 ± 5.0 Aa	74,163.4 ± 5876.7 Aa	0.46 ± 0.03 Ba
C2W1	8088.9 ± 348.9 Bb	88.5 ± 5.9 Ab	62,671.7 ± 1339.4 Bb	0.45 ± 0.01 Bb
C3W1	7493.0 ± 237.6 Bc	87.9 ± 2.1 Ac	54,187.5 ± 5616.6 Bc	0.51 ± 0.01 Ac
C1W2	6449.2 ± 260.4 Cb	61.3 ± 1.2 Bb	57,180.5 ± 2819.8 Cb	0.39 ± 0.01 Eb
C2W2	5111.6 ± 158.6 Dc	50.9 ± 0.3 Cc	43,150.4 ± 1640.4 Dc	0.44 ± 0.01 Db
C3W2	4224.2 ± 169.6 Ed	43.9 ± 3.7 Dd	25,719.9 ± 235.8 Ed	0.47 ± 0.02 Cd
C	*	*	*	*
W	*	*	*	*
C*W	NS	NS	NS	*

Note: The capital letters in the same column represent significant differences (*p* < 0.05) at different compaction levels under the same water condition. The small letters in the same column represent significant differences (*p* < 0.05) at different soil water status under the same compaction level. * indicates significant differences at *p* < 0.05. NS, not significant at *p* < 0.05.

## Data Availability

The data will be made available on request.

## References

[1] Hartemink A.E. (2008). Soils Are Back on the Global Agenda. Soil Use Manag..

[2] Gregorich E.G., McLaughlin N.B., Lapen D.R., Ma B.L., Rochette P. (2014). Soil Compaction, Both an Environmental and Agronomic Culprit: Increased Nitrous Oxide Emissions and Reduced Plant Nitrogen Uptake. Soil Sci. Soc. Am. J..

[3] Shah A.N., Tanveer M., Shahzad B., Yang G., Fahad S., Ali S., Bukhari M.A., Tung S.A., Hafeez A., Souliyanonh B. (2017). Soil Compaction Effects on Soil Health and Cropproductivity: An Overview. Environ. Sci. Pollut. Res..

[4] Batey T. (2009). Soil Compaction and Soil Management—A Review. Soil Use Manag..

[5] Hamza M.A., Anderson W.K. (2003). Responses of Soil Properties and Grain Yields to Deep Ripping and Gypsum Application in a Compacted Loamy Sand Soil Contrasted with a Sandy Clay Loam Soil in Western Australia. Aust. J. Agric. Res..

[6] Horn R., Fleige H. (2009). Risk Assessment of Subsoil Compaction for Arable Soils in Northwest Germany at Farm Scale. Soil Tillage Res..

[7] Correa J., Postma J.A., Watt M., Wojciechowski T. (2019). Soil Compaction and the Architectural Plasticity of Root Systems. J. Exp. Bot..

[8] Sun X., She D., Fei Y., Wang H., Gao L. (2021). Three-Dimensional Fractal Characteristics of Soil Pore Structure and Their Relationships with Hydraulic Parameters in Biochar-Amended Saline Soil. Soil Tillage Res..

[9] Pfeifer J., Faget M., Walter A., Blossfeld S., Fiorani F., Schurr U., Nagel K.A. (2014). Spring Barley Shows Dynamic Compensatory Root and Shoot Growth Responses When Exposed to Localised Soil Compaction and Fertilisation. Funct. Plant Biol..

[10] Lipiec J., Hatano R. (2003). Quantification of Compaction Effects on Soil Physical Properties and Crop Growth. Geoderma.

[11] Lipiec J., Horn R., Pietrusiewicz J., Siczek A. (2012). Effects of Soil Compaction on Root Elongation and Anatomy of Different Cereal Plant Species. Soil Tillage Res..

[12] Jin K., Shen J., Ashton R.W., White R.P., Dodd I.C., Phillips A.L., Parry M.A.J., Whalley W.R. (2015). The Effect of Impedance to Root Growth on Plant Architecture in Wheat. Plant Soil.

[13] Potocka I., Szymanowska-Pułka J. (2018). Morphological Responses of Plant Roots to Mechanical Stress. Ann. Bot..

[14] Bouwman L.A., Arts W.B.M. (2000). Effects of Soil Compaction on the Relationships between Nematodes, Grass Production and Soil Physical Properties. Appl. Soil. Ecol..

[15] Zhang X., Hua Z., Deng H. (2014). Effects of soil compaction stress on growth, quantity and quality of Scutellaria baicalensis. Soil. Fert. Sci. China.

[16] Whalley W.R., Clark L.J., Gowing D.J.G., Cope R.E., Lodge R.J., Leeds-Harrison P.B. (2006). Does Soil Strength Play a Role in Wheat Yield Losses Caused by Soil Drying?. Plant Soil.

[17] Wang X., Shen J., Hedden P., Phillips A.L., Thomas S.G., Ge Y., Ashton R.W., Whalley W.R. (2021). Wheat Growth Responses to Soil Mechanical Impedance Are Dependent on Phosphorus Supply. Soil Tillage Res..

[18] Yan M., Yang D., He Y., Ma Y., Zhang X., Wang Q., Gao J. (2024). Alfalfa Responses to Intensive Soil Compaction: Effects on Plant and Root Growth, Phytohormones and Internal Gene Expression. Plants.

[19] Shaheb R. (2020). A Study on the Effect of Tyre Inflation Pressure on Soil Properties, Growth and Yield of Maize and Soybean in Central Illinois. Ph.D. Thesis.

[20] Stenitzer E., Murer E. (2003). Impact of Soil Compaction upon Soil Water Balance and Maize Yield Estimated by the SIMWASER Model. Soil Tillage Res..

[21] Sun Y., Wang Y., Tong R. (2009). Effects of Soil Compaction Stress on Photosynthesis, Chlorophyll Fluorescence Parameters of Cucumber (*Cucumis sativus* L.) Leaves. J. Plant Nutr. Fertil..

[22] Ripley B.S., Gilbert M.E., Ibrahim D.G., Osborne C.P. (2007). Drought Constraints on C4 Photosynthesis: Stomatal and Metabolic Limitations in C3 and C4 Subspecies of Alloteropsis Semialata. J. Exp. Bot..

[23] Schlüter S., Weller U., Vogel H.-J. (2011). Soil-Structure Development Including Seasonal Dynamics in a Long-Term Fertilization Experiment. J. Plant Nutr. Soil Sci..

[24] Steponavičienė V., Bogužas V., Sinkevičienė A., Skinulienė L., Vaisvalavičius R., Sinkevičius A. (2022). Soil Water Capacity, Pore Size Distribution, and CO_2_ Emission in Different Soil Tillage Systems and Straw Retention. Plants.

[25] Bengough A.G., McKenzie B.M., Hallett P.D., Valentine T.A. (2011). Root Elongation, Water Stress, and Mechanical Impedance: A Review of Limiting Stresses and Beneficial Root Tip Traits. J. Exp. Bot..

[26] Grzesiak M.T., Janowiak F., Szczyrek P., Kaczanowska K., Ostrowska A., Rut G., Hura T., Rzepka A., Grzesiak S. (2016). Impact of Soil Compaction Stress Combined with Drought or Waterlogging on Physiological and Biochemical Markers in Two Maize Hybrids. Acta Physiol. Plant..

[27] Budhathoki S., Lamba J., Srivastava P., Williams C., Arriaga F., Karthikeyan K.G. (2022). Impact of Land Use and Tillage Practice on Soil Macropore Characteristics Inferred from X-Ray Computed Tomography. Catena.

[28] Colombi T., Braun S., Keller T., Walter A. (2017). Artificial Macropores Attract Crop Roots and Enhance Plant Productivity on Compacted Soils. Sci. Total Environ..

[29] Pires L.F., Roque W.L., Rosa J.A., Mooney S.J. (2019). 3D Analysis of the Soil Porous Architecture under Long Term Contrasting Management Systems by X-Ray Computed Tomography. Soil Tillage Res..

[30] Chen Y.L., Palta J., Clements J., Buirchell B., Siddique K.H.M., Rengel Z. (2014). Root Architecture Alteration of Narrow-Leafed Lupin and Wheat in Response to Soil Compaction. Field Crops Res..

[31] Wang Q., Li C., Li Q., Xue S. (2011). Effect of Soil Compaction on Spatio-Temporal Distribution and Activities in Maize Under Different Soil Types. Chin. Agric. Sci..

[32] Tubeileh A., Groleau-Renaud V., Plantureux S., Guckert A. (2003). Effect of Soil Compaction on Photosynthesis and Carbon Partitioning within a Maize–Soil System. Soil Tillage Res..

[33] Cambi M., Hoshika Y., Mariotti B., Paoletti E., Picchio R., Venanzi R., Marchi E. (2017). Compaction by a Forest Machine Affects Soil Quality and *Quercus robur* L. Seedling Performance in an Experimental Field. For. Ecol. Manag..

[34] Vincent C., Rowland D.L., Schaffer B. (2015). The Potential for Primed Acclimation in Papaya (*Carica papaya* L.): Determination of Critical Water Deficit Thresholds and Physiological Response Variables. Sci. Hortic..

[35] Leskovar D.I., Othman Y.A. (2021). Direct Seeding and Transplanting Influence Root Dynamics, Morpho-Physiology, Yield, and Head Quality of Globe Artichoke. Plants.

[36] Ren B., Zhang J., Dong S., Liu P., Zhao B. (2017). Regulations of 6-Benzyladenine (6-BA) on Leaf Ultrastructure and Photosynthetic Characteristics of Waterlogged Summer Maize. J. Plant Growth Regul..

[37] Chen B., Huang G., Lu X., Gu S., Wen W., Wang G., Chang W., Guo X., Zhao C. (2023). Prediction of Vertical Distribution of SPAD Values within Maize Canopy Based on Unmanned Aerial Vehicles Multispectral Imagery. Front. Plant Sci..

[38] Doube M., Kłosowski M.M., Arganda-Carreras I., Cordelières F.P., Dougherty R.P., Jackson J.S., Schmid B., Hutchinson J.R., Shefelbine S.J. (2010). BoneJ: Free and Extensible Bone Image Analysis in ImageJ. Bone.

[39] Larsbo M., Koestel J., Jarvis N. (2014). Relations between Macropore Network Characteristics and the Degree of Preferential Solute Transport. Hydrol. Earth Syst. Sci..

[40] Bribiesca E. (2000). A Measure of Compactness for 3D Shapes. Comput. Math. Appl..

[41] Wang M., Xu S., Kong C., Zhao Y., Shi X., Guo N. (2019). Assessing the Effects of Land Use Change from Rice to Vegetable on Soil Structural Quality Using X-Ray CT. Soil Tillage Res..

[42] Dal Ferro N., Charrier P., Morari F. (2013). Dual-Scale Micro-CT Assessment of Soil Structure in a Long-Term Fertilization Experiment. Geoderma.

[43] Pajor R., Falconer R., Hapca S., Otten W. (2010). Modelling and Quantifying the Effect of Heterogeneity in Soil Physical Conditions on Fungal Growth. Biogeosciences.

[44] Singh N., Kumar S., Udawatta R.P., Anderson S.H., de Jonge L.W., Katuwal S. (2021). X-Ray Micro-Computed Tomography Characterized Soil Pore Network as Influenced by Long-Term Application of Manure and Fertilizer. Geoderma.

